# Evaluation of Body Mass Index and Survival of Nasopharyngeal Carcinoma by Propensity-Matched Analysis: An Observational Case-Control Study

**DOI:** 10.1097/MD.0000000000002380

**Published:** 2016-01-15

**Authors:** Pu-Yun OuYang, Lu-Ning Zhang, Jie Tang, Xiao-Wen Lan, Yao Xiao, Yuan-Hong Gao, Jun Ma, Fang-Yun Xie

**Affiliations:** From the Department of Radiation Oncology, Sun Yat-sen University Cancer Center, State Key Laboratory of Oncology in South China, Collaborative Innovation Center for Cancer Medicine, Guangzhou, Guangdong, China.

## Abstract

The effect of pretreatment body mass index on survival of nasopharyngeal carcinoma remains contradictory.

All patients (N = 1778) underwent intensity-modulated radiotherapy with or without chemotherapy. Body mass index was categorized as underweight (<18.5 kg/m^2^), normal weight (18.5–22.9 kg/m^2^), overweight (22.9–27.5 kg/m^2^), and obesity (≥27.5 kg/m^2^). Propensity score matching method was used to identify patients with balanced characteristics and treatment regimen. Disease-specific survival (DSS), overall survival (OS), distant metastasis–free survival (DMFS), and locoregional relapse–free survival were estimated by Kaplan–Meier method and Cox regression.

Following propensity matching, 115 (underweight vs normal), 399 (overweight vs normal), and 93 (obese vs normal) pairs of patients were selected, respectively. In univariate analysis, underweight patients had inferior DSS/OS (*P* = 0.042) and DMFS (*P* = 0.025) while both overweight and obese patients showed similar survival across all the endpoints (*P* ≥ 0.098) to those with normal weight. In multivariate analysis, underweight remained predictive of poor DSS/OS (*P* = 0.044) and DMFS (*P* = 0.040), whereas overweight (*P* ≥ 0.124) or obesity (*P* ≥ 0.179) was not associated with any type of survival.

Underweight increased the risk of death and distant metastasis, whereas overweight or obese did not affect the survival of nasopharyngeal carcinoma. This provides support for early nutritional intervention during the long waiting time before treatment.

## INTRODUCTION

Nasopharyngeal carcinoma (NPC) is a distinct type of head and neck cancer, with particular etiology, epidemiology,^1^ symptoms, and therapeutic strategies.^2^ Despite the application of magnetic resonance imaging (MRI) and intensity-modulated radiotherapy (IMRT) and the assistance of chemotherapy regimens,^3,4^ the survival of patients with locoregionally advanced NPC remains unsatisfactory.^5^

It was reported that NPC patients with diverse body mass index (BMI) had different survival rates.^6–8^ But the results were quite contradictory. Two studies^6,7^ reported similar survival rates between patients with BMI <18.5 kg/m^2^ and 18.5–22.9 kg/m^2^, while another study^8^ observed adverse survival in case of BMI <18.5 kg/m^2^. Shen and colleagues^7^ found significant survival advantage of BMI ≥27.5 kg/m^2^ over 18.5–22.9 kg/m^2^, whereas this was the very reverse of the finding in the study by Huang and colleagues.^6^ These inconsistent findings were possibly related to the following issues. Shen and colleagues focused on the association of lifestyle factors (including BMI) and survival, but neglected important data on treatment.^7^ Huang and colleagues^6^ confined the analysis to patients with stage III–IV and took no account of other significant confounders, for example, smoking status, which is known to directly affect BMI^9–11^ and NPC survival.^12,13^ And the finding cannot apply to patients failing to meet the strict participant inclusion criteria in this randomized controlled trial.^6^ Hu and colleagues^8^ did not adopt the World Health Organization (WHO) criteria of BMI for Asian people and took BMI <18.5 kg/m^2^ as reference. Finally, the distribution of BMI changed as the increasing number of overweight and obese people in recent years.

As a potential, manageable prognostic factor, BMI shall be extremely valuable for suggesting early nutritional intervention and weight management during the long waiting time before the initial treatment. Therefore, we included 1778 NPC patients who underwent definitive IMRT with or without chemotherapy as recommended, to reevaluate the effect of BMI and survival using propensity score matching method and multivariate analysis.

## METHODS

### Patients

This study was approved by the institutional review board at Sun Yat-sen University Cancer Center. Formal consent is not required for this retrospective study, and individual informed consent was waived given the anonymous analysis of routine data. We included 1778 histologically proven and nonmetastatic NPC patients who underwent definitive IMRT with or without chemotherapy. Details of IMRT had been described previously.^5^ Most of patients with advanced stage received induction, concurrent, and adjuvant chemotherapy or combined treatment. Induction chemotherapy mainly consisted of cisplatin plus 5-fluorouracil, cisplatin plus taxane, or triplet of cisplatin plus 5-fluorouracil and taxane every 3 weeks for 2 to 3 cycles. Cisplatin-based concurrent chemotherapy was given weekly or every 3 weeks. Adjuvant chemotherapy was mainly delivered with 2 cycles of cisplatin plus 5-fluorouracil every 3 weeks. All patients were restaged according to the 2010 International Union against Cancer/ American Joint Committee on Cancer (UICC/AJCC) staging system for NPC.

Every 3 to 6 months during the first 3 years and every 6 to 12 months thereafter, patients were conventionally assessed by clinical symptoms, physical examinations, immunoglobulin A against viral capsid antigen (VCA-IgA) and early antigen (EA-IgA) of Epstein–Barr virus test, Epstein–Barr virus deoxyribonucleic acid copy number test (from 2009), and imaging methods, including MRI scan of the nasopharynx and neck, chest radiography, and/or computed tomography (CT), technetium-99m-methylene diphosphonate whole-body bone scan or CT/MRI scan of specific bones, and abdominal sonography and/or CT. Positron emission tomography-CT, biopsy, and/or fine-needle aspiration may be adopted as appropriate in doubtful cases of locoregional relapses or distant metastases. Patients with relapse, distant metastasis, or in persistent disease underwent salvage treatment including reirradiation, chemotherapy, and surgery.

### Statistical Analysis

WHO cut points for Asian people were used to categorize patients by BMI as underweight (<18.5 kg/m^2^), normal weight (18.5–22.9 kg/m^2^), overweight (22.9–27.5 kg/m^2^), and obese (≥27.5 kg/m^2^). In the lack of a randomized controlled trial, we used propensity score matching method to well balance characteristics across BMI and consequently reduce possible biases to a minimum in a retrospective analysis.^14^ Propensity scores were computed by logistic regression for each patient in both case (underweight, overweight, or obese) and control (normal weight) sets. We considered sex, age, smoking, drinking, histology, titers of VCA-IgA and EA-IgA, T-stage, N-stage, clinical stage, and chemotherapy regimens in this analysis. Patients in case and control sets were then matched without replacement at the equal ratio. Covariates balance between case and control sets was examined by *t* test (continuous variable), χ^2^ test, or Fisher exact test (categorical variable) as appropriate.

Disease-specific survival (DSS, the time from treatment to the death resulting from NPC or treatment complications), overall survival (OS, the time from treatment to the death from any cause), distant metastasis–free survival (DMFS, the time from treatment to the first distant metastasis), and locoregional relapse–free survival (LRFS, the time from treatment to the first locoregional relapse) were estimated using Kaplan–Meier methods and log-rank test. Hazard ratios (HRs) and 95% confidence intervals (CIs) were calculated with the Cox proportional hazards model.^15^ Multivariate analyses were performed using Cox proportional hazards model with enter method for BMI, T-stage and N-stage, and forward likelihood ratio method for other covariates.

All statistical analyses were performed using IBM SPSS Statistics version 22.0. Two-sided *P* values < 0.05 were considered to be significant.

## RESULTS

### Patients

Overall, 1778 patients were included in the study. Table 1 displayed the baseline characteristics. Respectively, 708 (39.8%), 123 (6.9%), 792 (44.5%), and 155 (8.7%) patients had normal weight, underweight, overweight, and obesity at the time of diagnosis. The average age at diagnosis, sex, smoking, drinking, and histological type were quite balanced among these patients. Compared with normal weight patients, underweight patients were more likely to be diagnosed with early T-stage, N-stage, and clinical stage and to avoid chemotherapy, whereas precisely the opposite happened to patients with overweight or obesity (all *P* ≤ 0.002). Additionally, overweight patients showed significantly higher titer of VCA-IgA (*P* = 0.015) and EA-IgA (*P* = 0.003) than those with normal weight.

**TABLE 1:**
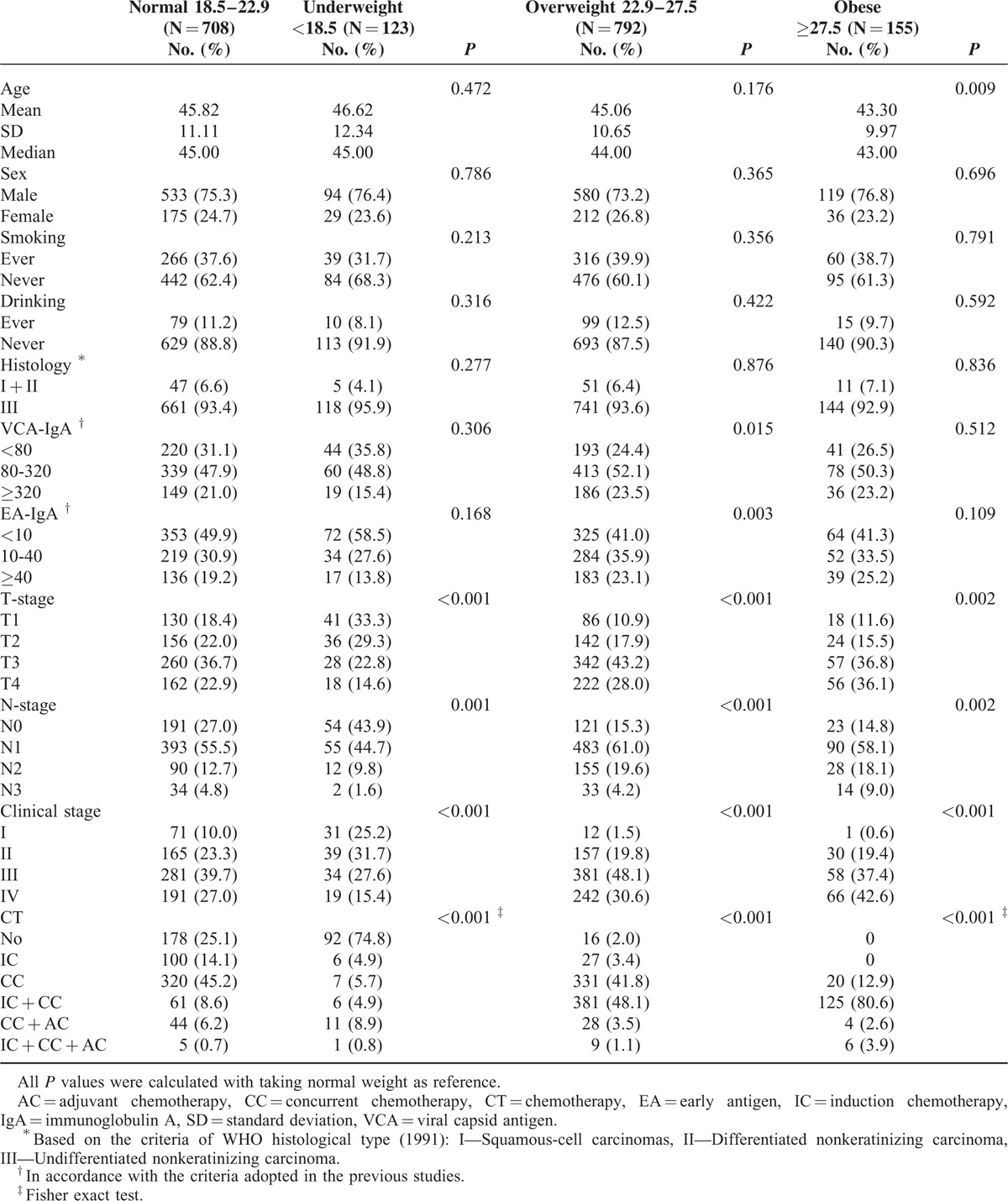
Baseline Characteristics of the Included 1778 Patients Before Propensity Score Matching

Following propensity score matching, 115 pairs (underweight vs normal), 399 pairs (overweight vs normal), and 93 pairs (obese vs normal) of patients were totally matched in the aspect of average age at diagnosis, sex, smoking, drinking, histological type, T-stage, N-stage, clinical stage, and chemotherapy regimens (Table 2). All subsequent analyses were based on the propensity-matched cohorts.

**TABLE 2:**
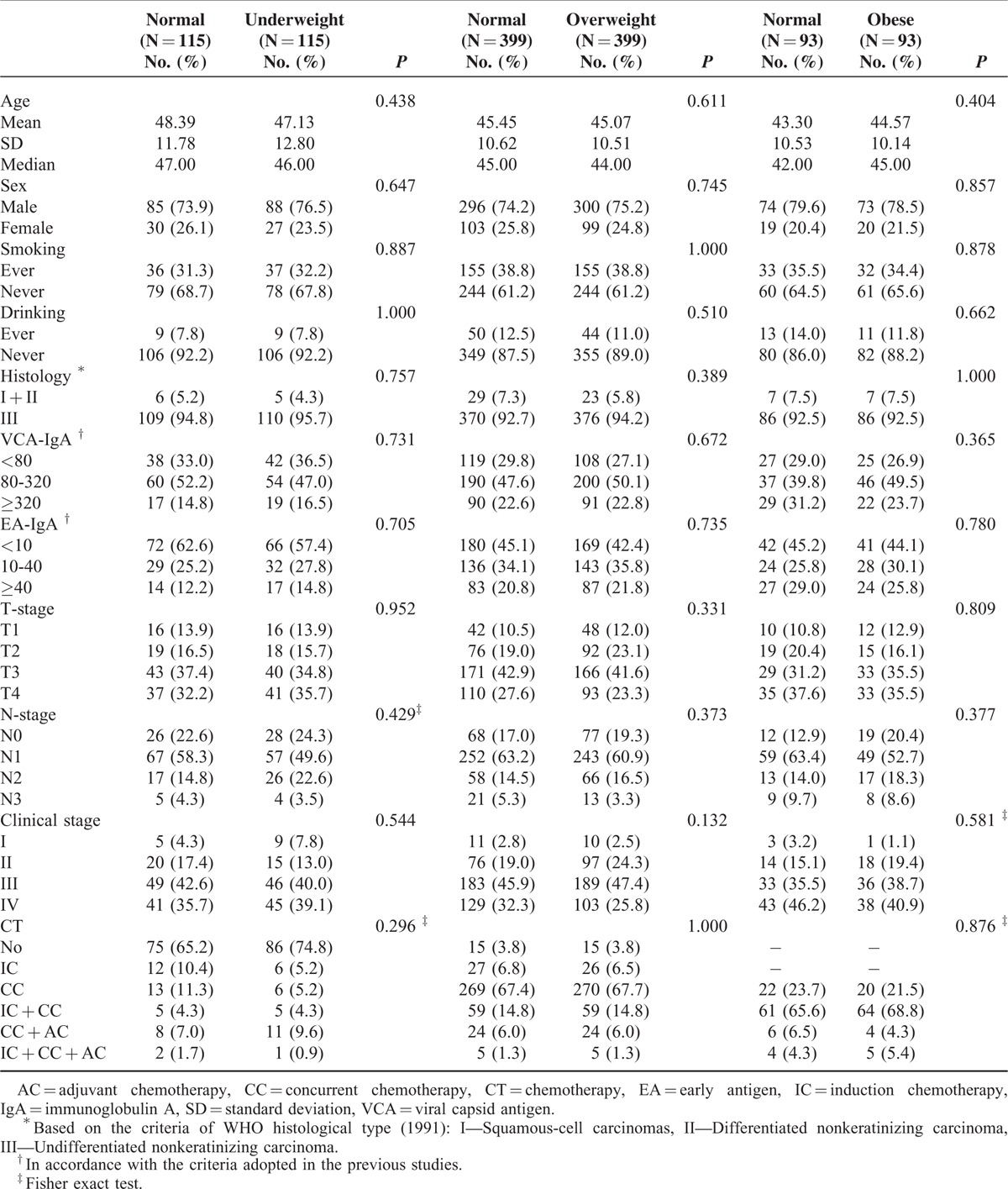
Baseline Characteristics in 3 Cohorts of Matched Pairs After Propensity Score Matching

### BMI and Survival

Interestingly, all the death events resulted from cancer or treatment complications. Thus, DSS was equal to OS in this study.

**FIGURE 1:**
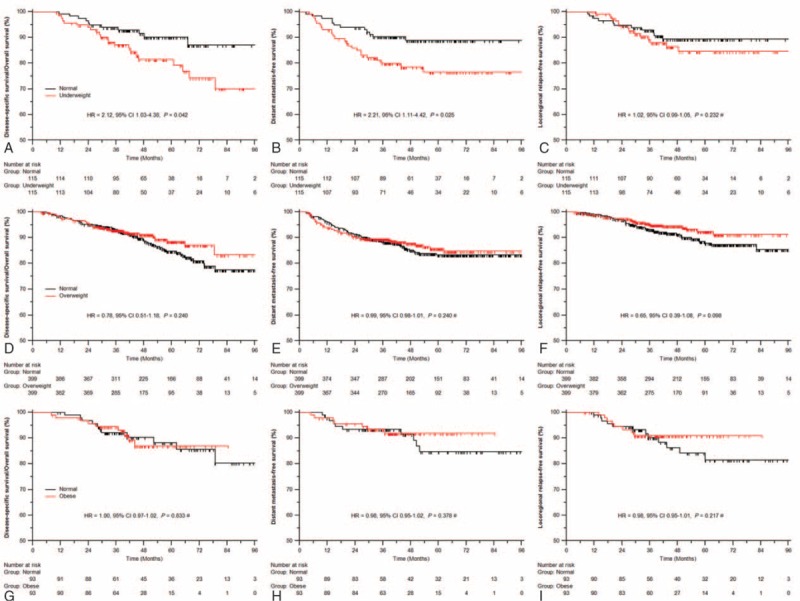
Comparison outcomes of survival: underweight versus normal weight (A–C), overweight versus normal weight (D–F), and obesity versus normal weight (G–I). ^∗^ Cox regression model with time-dependent covariates.

In the underweight versus normal weight cohort, the median follow-up was 47.9 months (10.2–106.7 months). Overall, the 4-year DSS/OS, DMFS, and LRFS rates were 81.5% versus 90.1% (*P* = 0.042), 78.5% versus 88.9% (*P* = 0.025), and 84.5% versus 89.4% (*P* = 0.232) for patients with underweight versus normal weight, respectively (Figure 1A–C). Compared with normal weight patients, underweight patients had 2.1-fold higher probability of death (*P* = 0.044) and distant metastasis (*P* = 0.040) but similar risk of locoregional relapse (*P* = 0.219) by multivariate analysis (Table 3).

**TABLE 3:**
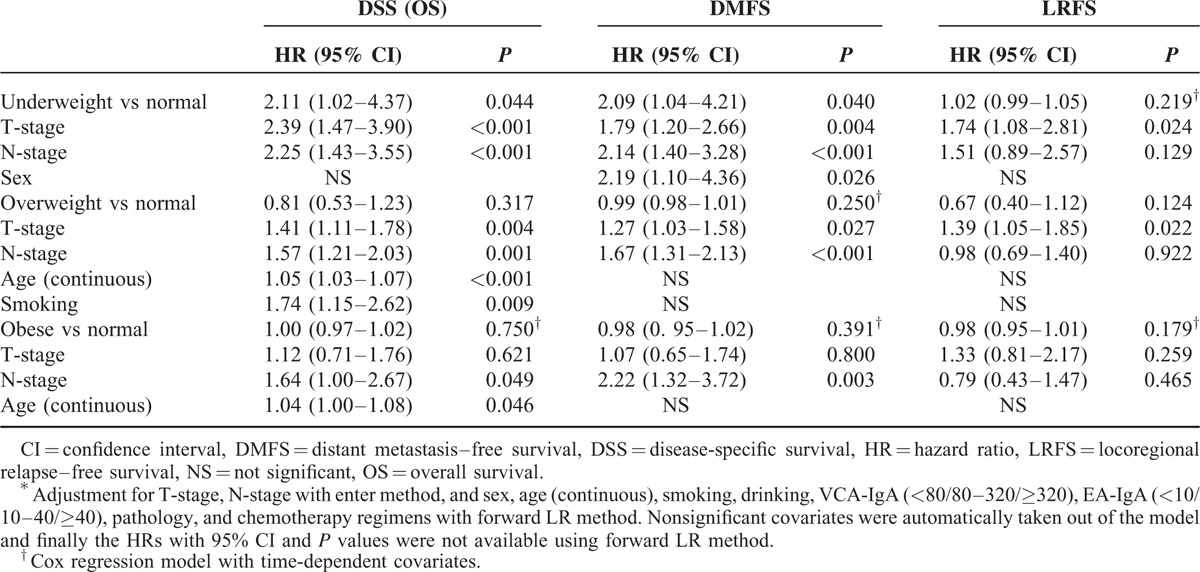
Summary of Important Prognostic Factors in Multivariate Analysis ^∗^

In the overweight versus normal weight cohort, the median follow-up was 48.2 months (3.3–105.7 months). Univariate analysis showed no significant differences in risk of death (4-year DSS/OS 91.2% vs 88.6%, *P* = 0.240), distant metastasis (87.8% vs 85.1%, *P* = 0.240), or locoregional relapse (93.9% vs 91.3%, *P* = 0.098) between overweight and normal weight patients (Figure 1D–F). In multivariate analyses, overweight was not significantly associated with any type of survival (all *P* ≥ 0.124) (Table 3).

In the obese versus normal weight cohort, the median follow-up was 42.0 months (8.0–105.7 months). Obese patients were found to be similar to those with normal weight in risk of death (3-year DSS/OS 93.0% vs 92.0%, *P* = 0.833), distant metastasis (91.8% vs 93.0%, *P* = 0.378), and locoregional relapse (91.0% vs 90.1%, *P* = 0.217) by univariate analysis (Figure 1G–I) and multivariate analysis (all *P* ≥ 0.179) (Table 3).

## DISCUSSION

As accumulating evidence demonstrated stronger association between obesity and mortality in never than ever smokers,^9–11^ it means that smoking can absolutely reduce the increase in relative mortality resulted from excess BMI. What is more, smoking is known to promote the development of NPC in population^16^ and increase the risk of treatment failure and mortality in NPC patients.^12,13^ So it is of particular importance to account for the confounding influence of smoking. Secondly, adiposity was found to accelerate the tumor growth and progression via insulin resistance, hyperinsulinemia, hyperglycemia, and chronic low-grade inflammation.^17^ Thus, the consequent on the interaction between excess BMI and tumor stage would cover the true survival differences across BMI if balancing tumor stage is failed. And similar interaction between BMI and chemotherapy may further increase the interference. Accordingly, the true prognostic impact of BMI on NPC survival cannot be exactly evaluated before completely balancing these factors.

In contrast to prior studies, our study included all patients receiving IMRT, fully balanced characteristics, and chemotherapy regimen using propensity matching method and further adjusted for these confounders with multivariate analysis. We found that underweight patients had 2-fold higher risk of death and distant metastasis than those with normal weight, whereas both overweight and obese patients were similar to those with normal weight across all the endpoints (DSS, OS, DMFS, and LRFS).

The mechanism by which underweight before treatment may lower NPC survival is not well understood. It is usually assumed that underweight is possibly associated with an advanced stage and an aggressive type of tumor. But this cannot completely explain the adverse survival for underweight NPC. This disease hardly hinders oral intake and causes severe weight loss on the whole, despite that certain patients possibly suffer weight loss owing to preclinical diseases. What is more important, we actually observed that underweight patients were more likely to be those with early stage NPC before propensity matching (Table 1). Further, the inferior survival for underweight patients was observed by comparison of underweight and normal weight patients with similar tumor stage and chemotherapy regimen after propensity score matching. So the intrinsic trait of underweight maybe works in a much more profound way. Underweight patients are often malnourished or even cachectic. Among these patients, the decrease of protein anabolism and caloric intake, coupled with the increase of protein catabolism, lipolysis, and resting energy expenditure, eventually causes impaired immunity and reduced survival.^18^ Additionally, the established influence of protein-energy malnutrition on immunity more likely results in the increased infectious toxicity and inflammation reaction in underweight patients.^19^ The induced and persisted high level of systemic inflammatory factors, such as tumor necrosis factor-α, and interleukin-6, can facilitate tumor cell proliferation and progression and enhance malignant properties.^20–22^ Finally, the malnutrition status of underweight patients may reduce chemotherapy response and increase chemotherapy toxicity.^23^

Overweight or even obese patients were reported to have higher survival rate than those with normal weight in prior studies,^6,7^ based on the hypothesis that the higher nutritional stocks of overweight or obese patients can help withstand weight loss during treatment. The fact is that high weight loss can significantly lower the survival rate for normal weight patients, but not for those with overweight or obesity.^24^ Overweight or obesity itself showed no protective effect on NPC survival. Inversely, adiposity in overweight and obese patients has been linked to increased circulating levels of insulin and insulin-like growth factor 1, which promote cell proliferation and inhibit apoptosis in NPC.^25^ And insulin-resistant adipocytes can activate macrophages to release proinflammatory mediators, including tumor necrosis factor-α and interleukin-6,^26^ which can facilitate tumor cell proliferation and progression and enhance malignant properties.^20–22^ That is, adiposity tends to promote NPC cell viability and progression in preclinical research, and we herein did note that obese patients were more likely to be diagnosed with advanced-stage disease (Table 1). Although tumor stage of normal and overweight/obese patients was balanced after propensity matching, adiposity can still continue exerting the effect of promoting cell viability and progression during the long course of chemoradiotherapy. Additionally, obesity and/or overweight have been associated with adverse survival in most cancers, such as the breast,^27,28^ pancreas,^29^ esophagus,^30^ colon, and rectum.^31,32^ Therefore, the comprehensive effect of midtreatment weight loss and adiposity may finally cause the similar survival between overweight/obese patients and those with normal weight in our study.

Midtreatment weight loss thus seemed to be a significant confounder. However, it is inappropriate to account for it in multivariate analysis, because midtreatment weight loss is unknown at the time of diagnosis. It would be actually helpless for the management of pretreatment weight if we found that overweight or obesity showed survival advantage over normal weight just among patients who suffered high weight loss during subsequent chemoradiotherapy. Moreover, this survival advantage derived from the decreased survival rate for normal weight patients who suffered high weight loss instead of the increased survival as a result of the protective effect of overweight or obese.^24^ So this observed advantage may disappear if normal weight patients maintain the weight till the end of treatment.

Despite that retrospective design of this study can weaken data collection, clinicopathologic and survival data were verified by review of individual patient records. Importantly, various confounders were totally balanced by propensity score matching, which provided the fairest comparison of survival for underweight, overweight, obese, and normal weight NPC patients.

It is a limitation that data on pretreatment weight loss were inaccurate or unavailable in the medical records, thus the influence was not included in our analysis; however, no study has demonstrated its prognostic effect in NPC. Owing to restaging according to the seventh edition of UICC/AJCC staging system, some patients in early stage received combined treatment of IMRT plus chemotherapy instead of IMRT alone. In addition, only 93 pairs of obese and normal weight patients were matched, which may lower the confidence of this comparison.

In conclusion, underweight patients showed inferior survival while both overweight and obese patients had similar survival to those with normal weight. Importantly, this large-scale propensity-matched study identifies underweight to be a manageable risk factor and provides support for early nutritional intervention during the long waiting time before the initial treatment.
